# Detection of other pathologies when utilising computer-assisted digital solutions for TB screening

**DOI:** 10.5588/ijtldopen.24.0428

**Published:** 2024-12-01

**Authors:** J. Sebastian, I.D. Olaru, A. Giannakis, M. Arentz, S.V. Kik, M. Ruhwald, S. Linsen, G. Günther, P. Wolf, F.J. Herth, T. Weber, C.M. Denkinger

**Affiliations:** ^1^ Division of Infectious Disease and Tropical Medicine, Heidelberg University Hospital, Heidelberg, Germany;; ^2^ Clinical Research Department, London School of Hygiene & Tropical Medicine, London, UK;; ^3^ Institute of Medical Microbiology, University of Münster, Münster, Germany;; ^4^ Diagnostic and Interventional Radiology, Heidelberg University Hospital, Heidelberg, Germany;; ^5^ Second Department of Radiology, University General Hospital “ATTIKON,” National and Kapodistrian University of Athens, Athens, Greece;; ^6^ FIND, Geneva, Switzerland;; ^7^ Department of Pulmonology and Allergology, Inselspital, Bern University Hospital, Bern, Switzerland;; ^8^ Department of Medical Sciences, Faculty of Health Sciences, University of Namibia, Windhoek, Namibia;; ^9^ Department for Pneumology and Critical Care Medicine, Thoraxklinik, University of Heidelberg, Heidelberg, Germany;; ^10^ German Center of Infection Research, Partner site Heidelberg, Germany.

**Keywords:** tuberculosis screening, non-communicable diseases, artificial intelligence, chest X-ray, CAD

## Abstract

**BACKGROUND:**

Computer-aided detection (CAD) tools for TB detection have the potential to enable screening programmes and reduce the diagnostic gap in settings where access to radiologists is limited. However, there are concerns that other common chest X-ray (CXR) abnormalities not due to TB may be missed.

**METHODS:**

We assessed the performance of three commercialised CAD tools (qXR, INSIGHT CXR and DrAID^TM^ TB XR) to detect common non-TB abnormalities against readings with a standardised annotation guide by an expert radiologist. More than 20 well-characterised diagnoses besides TB significant in TB high-burden countries were examined.

**RESULTS:**

The 517 CXRs included were deemed abnormal by the three CAD with a sensitivity of respectively 97% (95% CI 95–98), 94% (95% CI 91–95), and 87% (95% CI 84–90) for INSIGHT CXR, qXR, and DrAID. The CAD generally detected abnormalities in patients with critical diagnoses such as lung cancer or heart failure. Performance for detecting other abnormalities was variable.

**CONCLUSION:**

This study showed that the three CAD tools identified CXRs as abnormal when diseases other than TB were present. Our findings alleviate ethical concerns of missing abnormalities other than TB when using commercially available CAD for TB screening and show their potential broader applicability.

## RÉSUMÉ

**CONTEXTE:**

Les outils de détection assistée par ordinateur (CAD, pour l’anglais « *computer-aided detection* ») pour l’identification de la TB présentent la possibilité de faciliter les programmes de dépistage et de réduire l'écart de diagnostic dans les environnements où l'accès aux radiologues est restreint. Néanmoins, il existe des préoccupations quant à la non-détection d'autres anomalies fréquentes sur les radiographies thoraciques (CXR, pour l’anglais « *chest X-ray* ») qui ne sont pas liées à la TB.

**MÉTHODES:**

Nous avons analysé l'efficacité de trois outils de CAD (qXR, INSIGHT CXR et DrAIDTM TB XR) dans la détection d'anomalies fréquentes non liées à la TB, en les comparant à des interprétations réalisées selon un guide d'annotation standardisé par un radiologue spécialisé. Plus de 20 diagnostics clairement définis, en plus de la TB, ont été évalués dans des pays où la charge de TB est élevée.

**RÉSULTATS:**

Les 517 CXR incluses ont été considérées comme anormales par les trois systèmes d'aide à la décision (CAD), affichant des sensibilités respectives de 97% (IC à 95% 95–98), 94% (IC à 95% 91–95) et 87% (IC à 95% 84–90) pour INSIGHT CXR, qXR et DrAID. Le CAD a généralement réussi à identifier des anomalies chez les patients souffrant de diagnostics graves tels que le cancer du poumon ou l'insuffisance cardiaque. Les performances en matière de détection d'autres anomalies ont montré des résultats variables.

**CONCLUSION:**

Cette recherche a révélé que les trois outils de CAD détectaient les CXR comme étant anormales en présence de pathologies autres que la TB. Nos résultats atténuent les inquiétudes éthiques liées à l'absence d'anomalies autres que la TB lors de l'utilisation des CAD disponibles sur le marché pour le dépistage de la TB, et mettent en lumière leur potentiel d'application dans un cadre plus étendu.

As the importance of artificial intelligence (AI) continues to grow daily, its significance within medicine has also experienced a notable surge in recent years. This is particularly evident in the advancement of technologies designed to analyse and interpret radiological images.^1^ In the last years, many new computer-aided detection (CAD) tools based on deep learning have been developed to aid in different areas of radiology, such as the detection of pneumothorax after needle biopsy or aiding in the detection of lung cancer on chest X-ray (CXR).^2,3^ CAD tools especially designed for improving TB screening have been developed (https://www.ai4hlth.org/). These technologies have the potential to address several gaps preventing the optimal use of CXR for TB, such as long reading time and shortage of radiologists in high-TB burden countries.^4,5^

TB continues to be one of the leading causes of death from a single infectious agent worldwide, with around 1.3 million TB-related deaths estimated to have occurred in 2022 and over 4 million cases undiagnosed and untreated,^6^ with the gap having widened during the COVID-19 pandemic, setting the global TB goals for reductions in TB burden off track.^6^ Hence, the urgent need to close this gap with more effective screening programmes and better diagnostics is even more important than ever, especially considering the United Nations Sustainable Development Goals.^7^ In this context, the WHO recently recommended the implementation of CAD tools for TB screening for patients ≥15 years,^8^ following promising findings of CAD performance.^9–11^

However, with a wider scale-up of CAD tools as a screening tool for TB, there is a potential that CAD may be used in the absence of a human reader. Therefore, concerns have been raised about missing abnormalities in CXRs, other than TB, which require further assessment. Individuals evaluated for TB within screening programmes, especially those who are symptomatic, may have respiratory or cardiac conditions other than TB detectable by CXR.^12^ This would lead to missed opportunities to capture and advance these patients in the care cascade. The objectives of this study were to determine whether commercially available CAD tools can detect abnormalities not due to TB and to evaluate if critical abnormalities are missed.

## METHODS

### Study population and setting

The study was conducted in a low TB burden country (Germany with a TB incidence of 4.9/100,000 habitants in 2022)^13^ to minimise chances of including CXRs from patients with undiagnosed TB while ensuring that the abnormalities present were well characterised, and the diagnosis was confirmed through a comprehensive work-up. The study was conducted at the Heidelberg University Hospital (UKHD; Heidelberg, Germany) and its associated chest hospital, which specialises in the care of patients with respiratory conditions. The UKHD is Germany’s second largest university hospital, with 2585 clinical beds and over one million outpatient cases providing care to a wide catchment population.

To ensure a broad spectrum of different findings on CXRs, a multidisciplinary team with expertise in different medical fields (internal and respiratory medicine, infectious diseases, radiology) compiled a list of 20 clinically relevant diagnoses (Supplementary Table S1) associated with abnormalities on CXRs, that are considered of importance in high TB burden settings. All patients of at least 14 years of age with any preselected diagnoses and with an abnormal posterior-anterior CXR were eligible for inclusion. Patients with a history of TB or diagnosed with TB within 6 months after the CXR examination were excluded. Other reasons for exclusion were the X-rays not being in a Digital Imaging and Communications in Medicine (DICOM) format, medical devices visible on the CXR or bedside CXRs.

### Study procedures

International Statistical Classification of Diseases and Related Health Problems, Tenth Revision (ICD-10) codes associated with the preselected diagnoses were retrieved. The hospital databases were searched primarily consecutively for these codes from 2019 backwards, with every ICD-10 code provided until 2004 (except for SARS-CoV-2 pneumonia, where the search was conducted from 2020 forward). The goal was to select 30 CXRs for each diagnosis. CXRs from patients with different manifestations and disease severity were included consecutively, resulting in a broad spectrum of abnormalities. The CXRs were anonymised using a non-commercial DICOM anonymisation tool developed by the International Organization of Migration. These anonymised DICOM images were then uploaded to a secure server at FIND (Foundation for Innovative New Diagnostics; Geneva, Switzerland) for analysis with the CAD software. CXR interpretation was used as a reference and was performed by a board-certified expert radiologist using a standardised annotation guide developed for the study (Supplementary Table S2). The radiologist was blinded to the patient diagnoses and the CAD interpretation results.

### CAD tools evaluated

Three different commercially available CAD products were evaluated in this study: qXR v3.2.9 (qXR, qure.ai, Mumbai, India), INSIGHT CXR v3.1.4 (INSIGHT CXR, Lunit Inc., Seoul, South Korea) and DrAID^TM^ TB XR v1.0 (DrAID; VinBrain, Hanoi, Vietnam). These CAD tools were installed by the companies on separate virtual machines under the control of FIND. After successful installation, CAD companies were blocked access to these virtual machines to allow for an independent evaluation. FIND then processed all anonymised DICOM images with each CAD solution according to the manufacturer’s instructions.

All three CAD tools in this study are designed to identify several different findings (e.g. consolidation, nodule, fibrosis) beyond TB. An overview of all definitions for abnormalities by the developers can be found in the supplement (Supplementary Table S2).

As an output, all CAD software generate a report with probability scores for each abnormality, including TB, and a dichotomous output for the presence or absence of each abnormality, depending on the threshold. Supplementary Figure S1 contains sample outputs generated by the three CAD tools. Furthermore, they can flag CXRs as generally abnormal. For all three CAD tools, the companies recommend thresholds indicating the presence of an abnormality are used: for INSIGHT CXR (scores range from 0 to 100) and the proposed threshold was 15; for qXR (scores range from 0 to 1), this threshold was 0.5, and for DrAID (scores 0–1) thresholds varied between 0.4 and 0.90 depending on the abnormality.

### Statistical analysis

We reported categorical variables as counts and percentages and continuous variables as medians and interquartile ranges (IQRs). Sensitivity and specificities were calculated for each abnormality and all abnormal findings overall using the radiologist as a reference. 95% confidence intervals (CIs) using the Wilson method were computed. The statistical analysis was conducted in R v4.3.1 (R Computing, Vienna, Austria).

### Patient consent

This retrospective study uses routine patient records and does not require patient consent. The study protocol was approved by the ethics commission of the Heidelberg medical faculty, UKHD, Heidelberg, Germany (S-577/2021). The study was reported according to the Standards for Reporting of Diagnostic Accuracy Studies (STARD) guidelines for reporting on diagnostic accuracy studies.^14^

## RESULTS

In total, 517 patients were included. For COVID-19 pneumonia (16 CXR included), silicosis (*n* = 10), lung abscess (*n* = 6) or pleural thickening (*n* = 8), not enough abnormal CXRs were found. This was due to computed tomography (CT) scans being the predominant imaging method for the suspected diagnosis (e.g. COVID-19), CXRs not matching our inclusion criteria (e.g. pleural thickening) or only a small number of patients with the target condition present (e.g. silicosis, lung abscess). Among the 517 patients, the median age was 65 years (IQR 51–74), 326 (63.1%) were male, and 6 (1.2%) were people living with HIV (Table 1). Four CXRs were excluded and not replaced because they were recorded as abnormal in the medical records but considered normal by the expert radiologist. All 517 CXRs were successfully processed by all three CAD software.

**Table 1.** Patient and radiological characteristics.

Characteristic	*n* (%)
Age, years, median [IQR]	65 [51–74]
Male sex	326 (63.1)
HIV infection	6 (1.2)
Radiological abnormalities as determined by an expert radiologist	
Atelectasis	174 (33.7)
Calcification	16 (3.1)
Cardiomegaly	251 (48.5)
Cavitary lesions	4 (0.8)
Dilated hilar pulmonary arteries	31 (6.0)
Elevated hemidiaphragm	40 (7.7)
Emphysema	33 (6.4)
Fibrosing interstitial lung disease	2 (0.4)
Hilar abnormalities	79 (15.3)
Mass (>3 cm)	44 (8.5)
Mediastinal widening	134 (25.9)
Nodule (<3 cm)	77 (14.9)
Opacity	261 (50.5)
Pleural effusion	139 (26.9)
Pneumoperitoneum	0 (0)
Pneumothorax	30 (5.8)
Reticulonodular opacities	41 (7.9)
Scarring	101 (19.5)
Tracheal shift	45 (8.7)
Number of radiological abnormalities	
1	108 (20.9)
2	127 (24.6)
3	116 (22.4)
4	82 (15.9)
≥5	84 (16.2)

*Reasons for not including 30 CXR for all diagnoses were as follows: CT scans being preferred over CXR for some diagnoses, CXR not meeting our inclusion criteria or having a small patient population for the diagnosis in question.

IQR = interquartile range; CT = computed tomography; CXR = chest X-ray.

All (100%) of the CXRs had at least one abnormality (any), according to the expert radiologist and were therefore, by definition, considered abnormal, with a median of 3 abnormalities (IQR 2–4) per CXR image. The most common abnormalities reported by the radiologist were opacities in 261 (50.5%), cardiomegaly in 251 (48.5%), atelectasis in 174 (33.7%), pleural effusion in 139 (26.7%), and mediastinal widening in 134 (25.9%).

**Figure 1.**
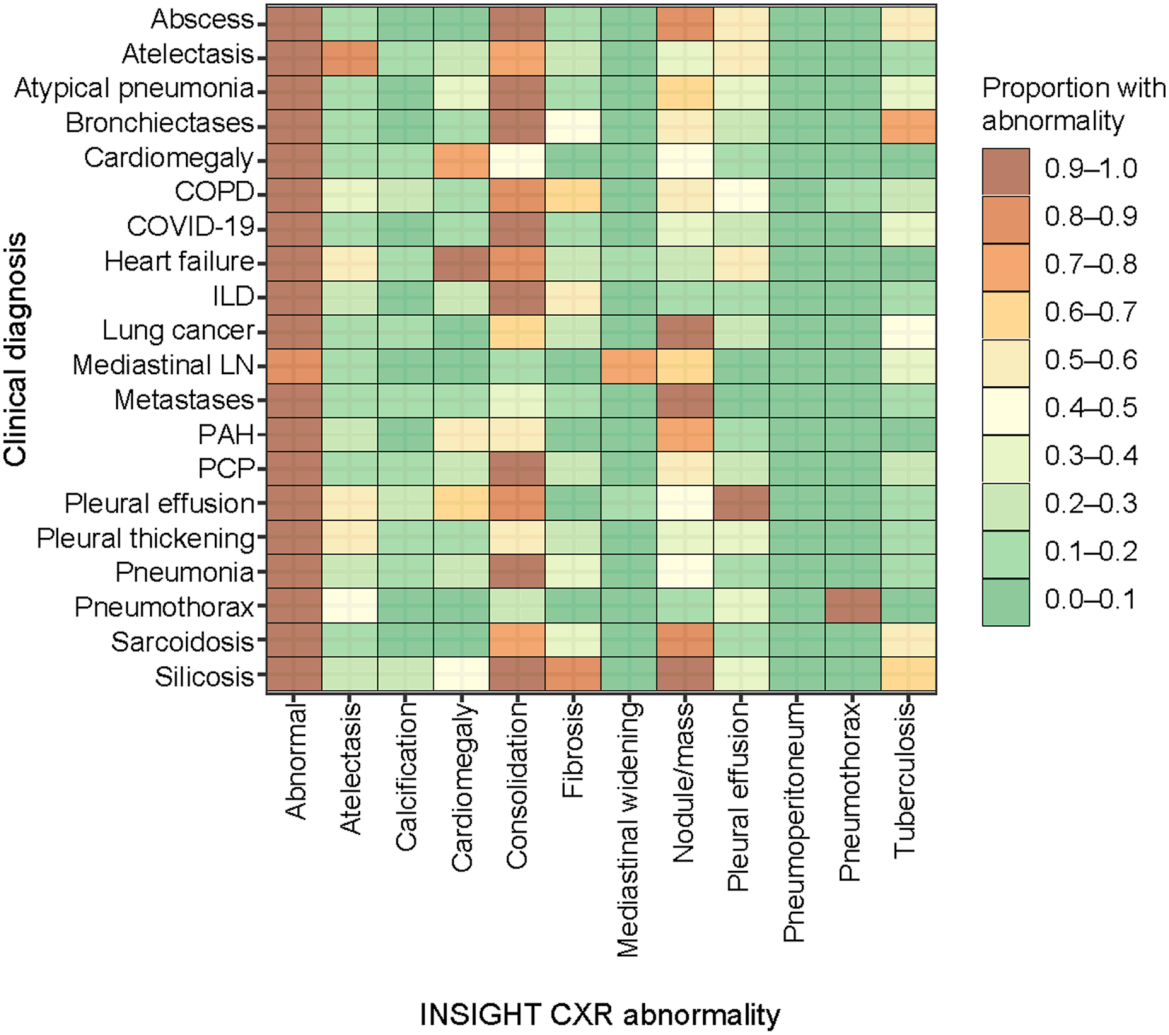
Distribution of abnormalities according to clinical diagnoses using INSIGHT CXR. Abnormalities are considered presented at abnormality scores above 15. COPD = chronic obstructive pulmonary disease; ILD = interstitial lung disease; LN = lymphadenopathy; PAH = pulmonary arterial hypertension; PCP = *Pneumocystis jirovecii* pneumonia; CXR = chest X-ray.

**Figure 2.**
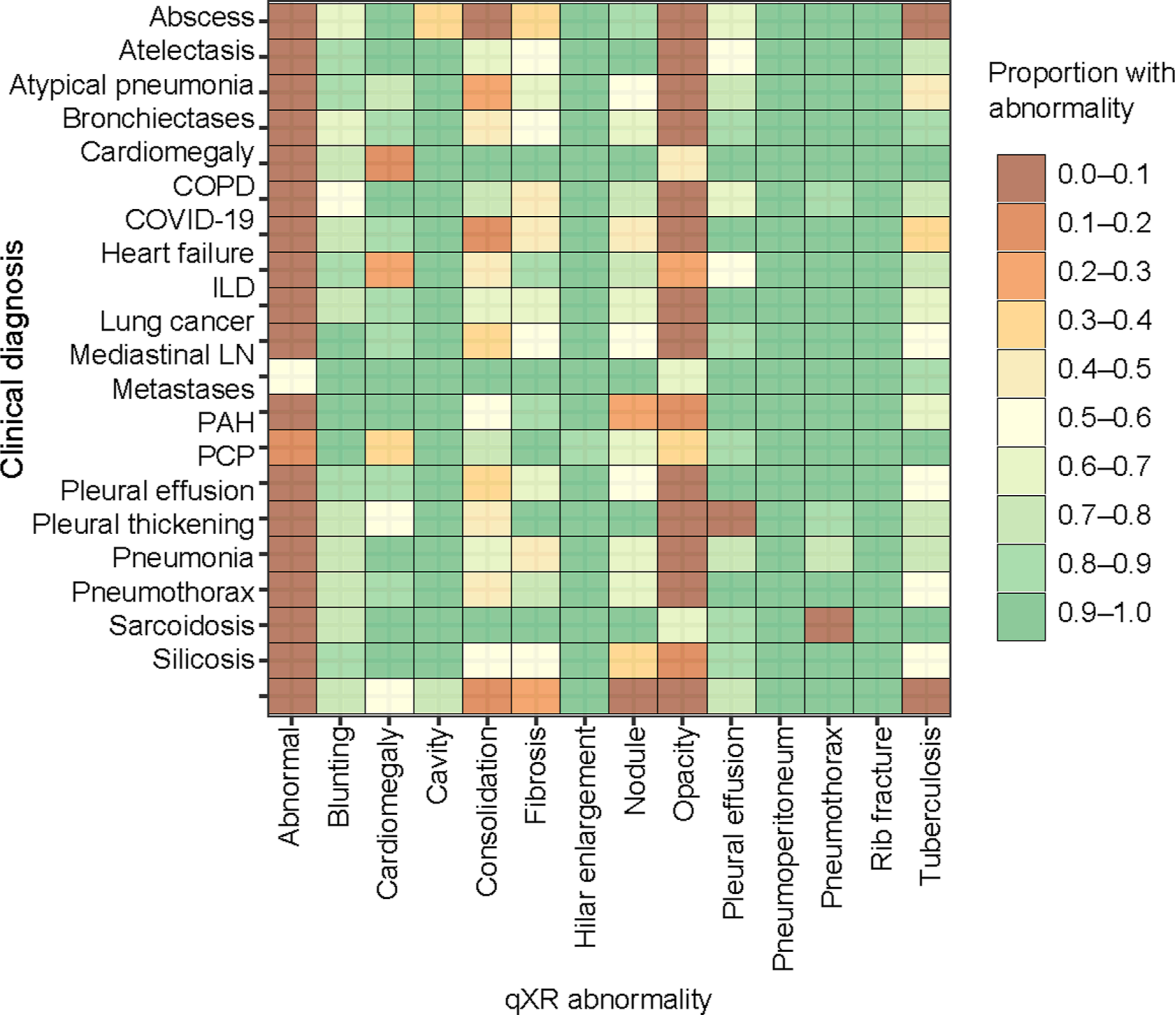
Distribution of abnormalities according to clinical diagnoses using qXR. Abnormalities are considered present at scores above 0.5. COPD = chronic obstructive pulmonary disease; ILD = interstitial lung disease; LN = lymphadenopathy; PAH = pulmonary arterial hypertension; PCP = *Pneumocystis jirovecii* pneumonia.

**Figure 3.**
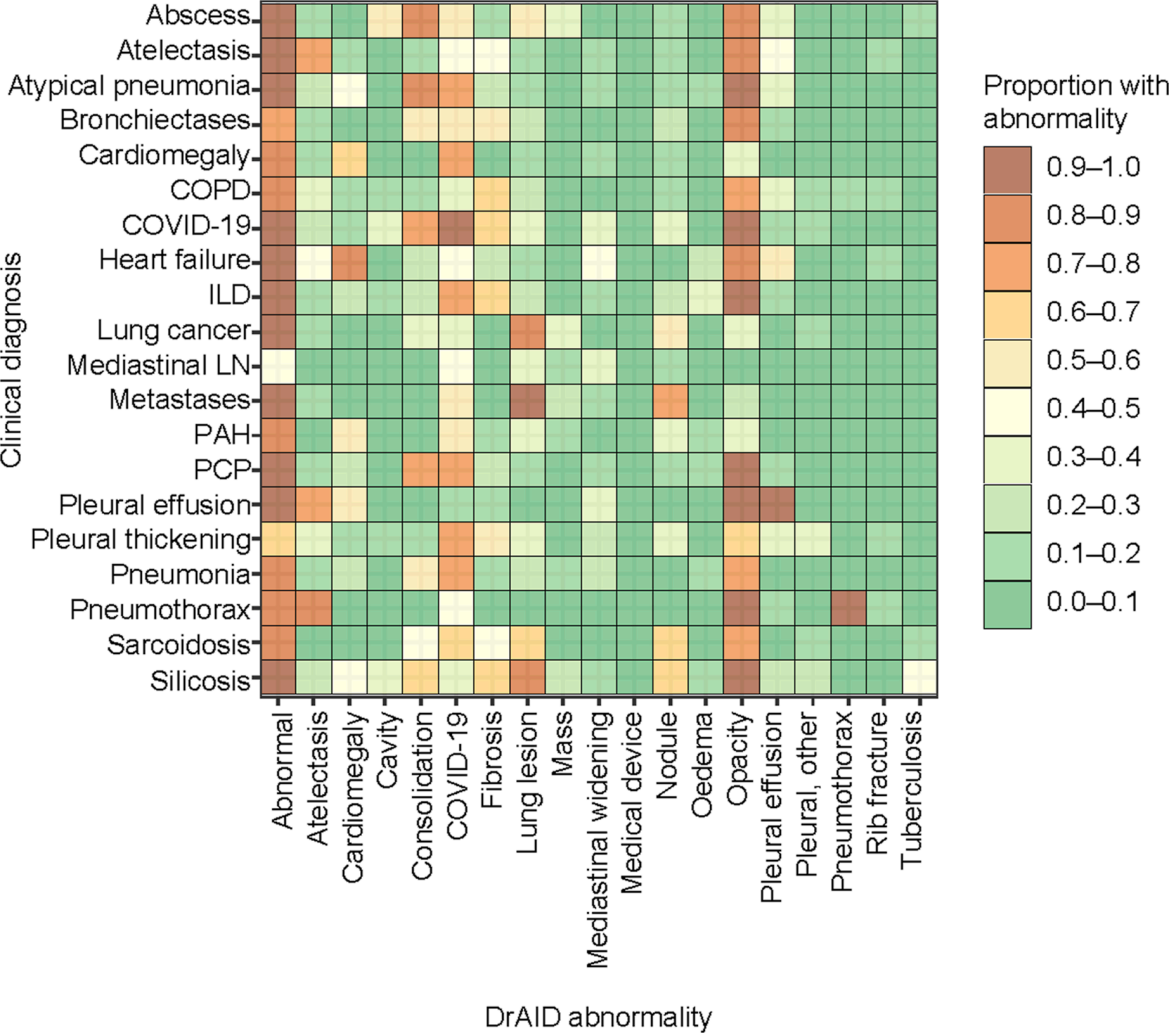
Distribution of abnormalities according to clinical diagnoses using DrAID. Abnormalities are considered present at scores above 0.4 for TB, 0.5 for abnormal medical device, above 0.55 for COVID-19; above 0.60 for oedema and pleural effusion, above 0.65 for cavity; above 0.70 for atelectasis, lung lesion, mass, nodule and opacity; above 0.80 for cardiomegaly and rib fracture; above 0.85 for consolidation, fibrosis, and pneumothorax, above 0.9 for other pleural findings. COPD = chronic obstructive pulmonary disease; ILD = interstitial lung disease; LN = lymphadenopathy; PAH = pulmonary arterial hypertension; PCP = *Pneumocystis jirovecii* pneumonia.

The three CAD systems deemed a CXR abnormal with a sensitivity of 97% (95% CI 95–98), 94% (95% CI 91–95), and 87% (95% CI 84–90) for INSIGHT CXR, qXR, and DrAID, respectively (Table 2). The distribution and overlap in scores for the three CAD according to the clinical diagnoses are shown in Figures 1–3, Supplementary Figures S2–S6, and Supplementary Tables S3 and S4.

**Table 2.** Performance of INSIGHT CXR, qXR and DrAID in detecting specific abnormalities using the interpretation by radiologist as reference standard.

Abnormality	Prevalence *n/N*	INSIGHT CXR (Lunit Inc., Seoul, South Korea)		qXR (qure.ai, Mumbai, India)		DrAID (VinBrain, Hanoi, Vietnam)	
		Sensitivity % (95% CI)	Specificity % (95% CI)	Sensitivity % (95% CI)	Specificity % (95% CI)	Sensitivity % (95% CI)	Specificity % (95% CI)
Total^*^	517/517	97 (95–98)	—	94 (91–95)	—	87 (84–90)	—
TB	0/517	—	76 (72–79)	—	71 (67–75)	—	98 (96–99)
Atelectasis	174/517	56 (49–63)	87 (83–90)	—	—	25 (20–31)	71 (66–77)
Calcification	16/517	56 (33–77)	92 (89–94)	—	—	—	—
Cardiomegaly	251/517	55 (49–61)	97 (95–99)	47 (41–53)	100 (99–100)	51 (45–58)	100 (99–100)
Cavity	4/517	—	—	100 (51–100)	98 (96–99)	75 (30–95)	94 (92–96)
Fibrosis	101/517	64 (55–73)	87 (83–89)	60 (51–69)	81 (77–85)	66 (57–75)	81 (77–84)
Mediastinal widening	134/517	29 (22–37)	99 (98–100)	—	—	44 (36–52)	96 (93–97)
Nodule	77/517	83 (75–89)^†^	55 (50–59)^†^	70 (59–79)	77 (73–81)	69 (58–78)	84 (80–87)
Opacity	261/517	97 (94–98)	54 (48–60)	98 (95–99)	32 (27–38)	90 (85–93)	54 (47–60)
Pleural effusion	139/517	76 (69–83)	88 (84–91)	58 (50–66)	96 (93–97)	65 (57–73)	93 (90–95)
Pneumothorax	30/517	100 (89–100)	99 (98–100)	93 (79–98)	96 (94–98)	97 (83–99)	99 (97–99)

* All CXRs included in the study were abnormal.

† Nodule and mass combined.

CXR = chest X-ray; CI = confidence interval.

The three CADs attributed abnormalities as possibly associated with TB in 126 (24.4%) patients using INSIGHT CXR, in 150 (29.0%) using qXR and in 12 (2.3%) using DrAID, resulting in specificity estimates of 76% (95% CI 72–79) for INSIGHT CXR, 71% (95% CI 67–75) for qXR and 98% (95% CI 96–99) for DrAID (Table 2). TB was frequently called in CXRs from individuals with bronchiectases (70.0%), silicosis (60.0%), sarcoidosis (57.0%) and lung abscesses (50.0%) for INSIGHT CXR. For qXRs, the most common conditions were lung abscesses (100.0%), silicosis (90.0%), COVID-19 pneumonia (63.0%) and atypical pneumonia (50.0%). Of the 12 cases that were categorised as TB using DrAID, 4 were silicosis cases (40.0%), and 3 were sarcoidosis (10.0%). An overview of all diagnoses flagged as TB by the CAD tools can be found in Supplementary Table S5.

### Detection of critical abnormalities

qXR had a sensitivity of 70% (95% CI 59–79) for the detection of nodules and a specificity of 77% (95% CI 73–81). INSIGHT CXR did not call nodules and masses separately but flagged both and had a sensitivity of 83% (95% CI 75–89) and a specificity of 55% (95% CI 50–59) for nodules and masses combined, as ascertained by the expert radiologist. DrAID had a sensitivity of 69% (95% CI 58–78) and a specificity of 84% (95% CI 80–87) for detecting nodules.

Of the 59 CXRs from patients with lung cancer or pulmonary metastases, respectively 2 (3.4%), 1 (1.7%), and 5 (8.5%) were considered normal using INSIGHT CXR, qXR, and DrAID (Supplementary Table S4). Of the six CXRs from patients with pulmonary abscesses, all were identified as abnormal by the three CAD systems. The radiologist identified post-inflammatory scarring in 101 CXRs. Of these, the INSIGHT CXR system classified 2 as normal and 65 as fibrotic; qXR found 1 normal and 61 with fibrosis; DrAID classified 8 as normal and 65 with fibrosis. In cases confirmed as fibrotic by the radiologist, the CAD systems reported TB in 32, 40, and 6 cases for INSIGHT CXR, qXR, and DrAID, respectively. Out of 30 patients diagnosed with heart failure, 29 were found by the radiologist to have cardiomegaly and/or pleural effusion. Both INSIGHT and DrAID detected these findings in all identified cases, whereas qXR identified them in only 26 of the 29 patients.

Among the 30 patients diagnosed with *Pneumocystis jirovecii* pneumonia, all CXRs were reported as abnormal by INSIGHT, and 29 by qXR and DrAID. For INSIGHT CXR, consolidation was detected in 29/30 CXRs; the one without consolidation had a nodule detected. For qXR, all 29/30 were reported as having opacities, while in 28/30, opacities were detected using DrAID.

## DISCUSSION

In this study, we assessed the performance of three CAD software programmes developed for TB in 517 patients with clinically relevant non-TB pathologies on CXR. Our findings suggest that CAD tools rarely call CXRs with clinically relevant abnormalities normal. The three different CAD tools correctly identified 87% or more of CXRs as abnormal. Overall, the best-performing CAD software for detecting the presence of any abnormality was INSIGHT CXR (97%, 95% CI 95–98), while DrAID performed the least well (87%, 95% CI 84–90). This alleviates ethical concerns about the widespread use of CAD, which is missing other clinically highly relevant diagnoses. The detection of other abnormalities not suggestive of TB would enable further assessment by a human reader to ensure that adequate care is provided.

As large-scale active case-finding strategies are needed to improve TB diagnosis and decrease community transmission,^15^ CAD tools are thus particularly attractive because they can be used as the initial screen for TB, with abnormal findings further referred for human review. CAD tools can screen thousands of individuals daily at scale, which is critical for high-burden settings where trained healthcare workers are scarce.^16^ In a TB screening scenario, where individuals from communities or risk groups are assessed, missed referral opportunities for people with abnormal findings for reasons other than TB can lead to missed opportunities for an early diagnosis (e.g. for malignancies), which is likely to be associated with worse outcomes, or with persisting symptoms impairing quality of life in those who have not yet sought care (e.g. heart failure). The consequences can be even more severe in a triage scenario, where people are actively seeking care for their symptoms and who, in the absence of further work-up and appropriate treatment, may not be able to re-present for care.

Certain abnormalities, such as pneumothorax and opacities, were identified well by all the CAD software (≥93% or 90% sensitivity, respectively), and findings were relatively consistent across the three products. While others were less well called out, e.g. cardiomegaly or pleural effusion (≤55% and 76% sensitivity, respectively). Critical lesions from patients with pulmonary cancer or metastases were rarely missed. Considering that more than 80% of the world’s people aged at least 60 years will be living in low- and middle-income countries by 2030, non-communicable diseases such as malignancies and chronic heart diseases are expected to increase further, and the ability of screening programmes for TB to ensure that these patients are entering the care cascade will become even more relevant.^17^ The vast majority of CXRs from patients with congestive heart failure, interstitial lung disease, chronic obstructive lung disease or lung cancer were considered abnormal. This was also the case for patients with acute infections such as pneumonia and lung abscesses.

Using CAD in addition to human reading can also be particularly beneficial when CXRs are read by less experienced radiologists and other healthcare workers interpreting CXR.^18^ A study has shown improved sensitivity and specificity for the detection of nodules on CXRs when compared to unaided reading. The increase was greater for less experienced readers. Furthermore, CAD alone performed better than radiologists for detecting nodules, irrespective of the level of training.^19^ CAD tools may improve access to diagnosis for a wide range of abnormalities in settings where radiologists are few or absent. Improved sensitivity in comparison to an expert radiologist and scenarios with assisted reading has been shown for other conditions, such as detecting pulmonary infiltrates in febrile neutropenia or assisting radiologists in improving their performance.^20^ CAD-assisted reading can also reduce variability between radiologist readings, increasing consistency.^20^

Although none of the CXRs were from patients diagnosed with TB, the INSIGHT CXR system suggested TB in one-quarter of this selected patient dataset, while qXR suggested it in nearly one-third. In contrast, DrAID only considered 2% of these CXRs compatible with a TB diagnosis. Overall specificity for TB detection in this selected population using the manufacturers’ proposed thresholds exceeded 70% for all CAD tools.^21^ This is not surprising given that many of the selected diagnoses have CXR findings that can overlap with TB (e.g. silicosis, cancer, abscess). Nevertheless, it is reassuring that in this challenging population, the specificity of the CADs still exceeded 70%.

This study has several strengths. It directly compares three different CAD tools across a wide range of diagnoses and associated CXR abnormalities. Also, given the selection of our experienced multidisciplinary team, a wide range of abnormalities relevant to pathologies in countries with high TB burdens were included. These diagnoses were established following comprehensive investigations available in a tertiary referral hospital from a high-resource setting. We selected, where possible, 30 CXR per pathology as well as a representative spectrum of findings for each clinical diagnosis to ensure that a broad range of abnormalities are included. Furthermore, all CXRs were thoroughly characterised and annotated by an experienced senior radiologist to ensure consistency of interpretations.

However, this study was also limited by the retrospective data collection, using CXRs from a single, highly specialised referral centre specifically selected for pathologies and thus does not reflect the population of patients seeking care in routine outpatient clinics or lower-level hospitals. In addition, results for rarely detected pathologies or diagnoses where only a few CXRs could be included (such as calcifications or lung abscesses) should be interpreted with caution due to the limited sample size. Also, CXRs were read by a single radiologist. While the radiologist was unaware of the diagnosis, the presence of an abnormality was a given. The specificity for the detection of abnormalities compatible with a TB diagnosis may have been underestimated as the radiologist was aware that none of the CXRs included were from patients with TB. Our study population, predominantly of European origin, had a median age of 65 years, making it notably distinct and considerably older than the populations expected to undergo screening and triage for TB in low- and middle-income countries. Lastly, newer software versions are being developed for all three CAD tools, which might show different performances.

Of note is that not all CAD tools reported the same radiological abnormalities. Additionally, there may be differences in how the CAD tools defined specific abnormalities and how the algorithms were developed, which may affect comparisons between the algorithms. Specifically, the developers may have used different reference standards to train their products, and CXR may have originated from various populations. Overall estimates may also have been affected by the strategy used for inclusion in the study (target number of CXR for selected diagnoses), which does reflect the distribution of these conditions in the population.

As expected, the CAD tools, since they are developed for TB screening, performed less well in patients with conditions which have similar radiological findings to those detected in patients with TB, such as bronchiectasis, lung abscesses, sarcoidosis and silicosis. Although we did not include patients with previous TB, fibrotic changes due to other conditions were relatively common.

In conclusion, our study showed that the three CAD tools developed and used in TB screening can detect other abnormalities than TB with a high rate of detection between 87% and 97% and critical abnormalities were rarely overlooked. Our findings alleviate possible ethical concerns of missing other abnormalities when using CAD for TB screening and also show a potential broader applicability for CAD. Nevertheless, limitations of the CADs are to be considered, such as over-calling abnormalities such as TB. While this is a problem also with human readers, it could lead to overdiagnosis and overtreatment of TB if the diagnosis is not confirmed with microbiological tests. Nevertheless, the benefits of testing at scale using CAD are very likely to largely outweigh the risks and lead to improved case detection and control for TB and to further investigations and management of patients with non-TB CXR abnormalities.

## Supplementary Material


