# Supplementary material for: Detection of other pathologies when utilising computer-assisted digital solutions for TB screening
Source: IJTLD Open. 2024 Dec 1;1(12):533–9. doi: 10.5588/ijtldopen.24.0428 (PMC11636493; doi:10.5588/ijtldopen.24.0428)
Supplement: Supplementary file 1 [file ijtldopen24-0428_supplementarydata1.pdf]

http://dx.doi.org/10.5588/ijtldopen.24.0428

Detection of other pathologies when utilising computer-assisted digital solutions for TB screening

Supplementary Data

Article title: Detection of other pathologies when utilizing computer-assisted digital radiography tools developed for tuberculosis screening

Table of Contents

Supplementary Table S1. ICD-10 codes used for X-ray selection 2

Supplementary Table S2. Overview of the definitions used for abnormality classification........ 3

Supplementary Table S3. Overview of diagnoses called as abnormal by the three CAD tools 7

Supplementary Table S4. Overview of detected abnormalities and scores for the three CAD 8

Supplementary Table S5. Tuberculosis called by CAD in CXRs from individuals with another clinical diagnosis 9

Supplementary Figure S1. Example X-rays for different pathologies using the three CAD systems 10

Supplementary Figure S2. Distribution of abnormality scores for INSIGHT CXR........ 11

Supplementary Figure S3. Distribution of abnormality scores for qXR........ 12

Supplementary Figure S4. Distribution of abnormality scores for DrAID 13

Supplementary Figure S5. Diagrams representing the overlap in reported findings for the three CAD technologies. 14

Supplementary Figure S6. Receiver operator curves for the performance of the three CAD technologies in detecting different abnormalities........ 16

Supplementary Table S1. ICD-10 codes used for X-ray selection

**Diagnosis**	**Number of X-rays included (%)**	**ICD-10 codes**
Atelectasis	30 (5.8)	J98.6
Atypical pneumonia	30 (5.8)	J10.0, J12.0/2/3/8
Bronchiectasis	30 (5.8)	J47
Cardiomegaly	30 (5.8)	I42.0/6
COPD	30 (5.8)	J43, J44
COVID-19 pneumonia	16 (3.1)	J12.8, U07.1
Interstitial lung disease	30 (5.8)	J84.1
Heart failure	30 (5.8)	I50.13, I11.00
Lung abscess	6 (1.2)	J85.0/1/2
Lung cancer	30 (5.8)	C34.0/1/2/3/8/9
Mediastinal lymphadenopathy	29 (5.6)	R59.0, R59.1, C81.1
Metastases	29 (5.6)	C78.0
Pleural effusion	30 (5.8)	J90
Pleural thickening	8 (1.5)	J92.-, J94.0, J94.1, J94.2, J94.8, J94.9, J94.-
Pneumocystis jirovecii pneumonia	30 (5.8)	B48.5
Pneumonia	29 (5.6)	J13, J14, J15, J16.0
Pneumothorax	30 (5.8)	J93.0/1
Pulmonary arterial hypertension	30 (5.8)	I27.0
Sarcoidosis	30 (5.8)	D86.0/1/3
Silicosis	10 (1.9)	J60, J61

Supplementary Table S2. Overview of the definitions used for abnormality classification

Abnormality	Radiologist	INSIGHT CXR	qXR	DrAID
Abnormal/ Normal	Abnormal	Any suspicious areas for radiologic findings detected in the Chest X-ray including 10 findings (atelectasis, calcification, cardiomegaly, consolidation, fibrosis, mediastinal widening, nodule, pneumothorax, pleural effusion and pneumoperitoneum) and TB	Any suspected abnormality in the Chest X-ray including but not limited to those listed below	No abnormalities detected on the chest X-ray.
Atelectasis	Atelectasis	Linear or wedge-shaped densely increased opacity with sharp demarcation, accompanied with volume decrease. Mostly targeted at subsegmental atelectasis. AI detects other types of collapse as well such as lobar collapse (labelled asatelectasis)	Evidence of lung collapse or volume loss due to reduced inflation of all or part of the lung	Lung atelectasis, lobar atelectasis, and segmental atelectasis.
Blunting	-	-	Blunting of either costo-phrenic angle, indicating the presence of a pleural effusion or pleural thickening	-
Calcification	Calcification (in the lung)	A highly dense nodular lesion suggestive of calcification or calcified granuloma in the lung.<br>Should not include atherosclerotic calcification shown at aorta and coronary artery. Calcified lesions shown in the areas other than chest such as abdomen, neck, spine must be disregarded as well.	-	-
Cardiomegaly	Cardiomegaly (cardiothoracic ratio > 0,5)	A significantly enlarged cardiac silhouette. Cardiothoracic ratio of 0.5 is not used as a criterion for deciding cardiomegaly. Radiologists have different opinions on borderline/mild cardiomegaly, and the aim of this task is to only mark the only evident ones. Therefore, the ones that are at the borderline (e.g., ratio = 0.52) will not be marked.	Increased heart size; cardio-thoracic ratio > 0.5	If postero-anterior view evaluate adequacy of inspiration (9 dorsal ribs, 6 ventral ribs) clearly shows heart contour on both sides, then uniformly label enlargement of the cardiac silhouette when cardiothoracic ratio is >0.5.
Cavity	Cavity + Nodule<br>Cavity + Mass	-	A gas-filled space with or without irregular margins, seen as a lucency or low attenuation	Cavitation is defined as gas-filled spaces in lung parenchyma, surrounded by an over-1-

	Cavity + Opacity		area, within pulmonary consolidation, a mass, or a nodule.	mm-thick wall (less-than-1-mm-thick walls are called air bubbles), with or without air crescent sign, air-fluid level, etc.
Consolidation		Homogeneously increased opacity with ill-defined margins, without volume decrease. Includes non-specific opacities shown in lung parenchyma. Different levels of ground glass opacification, patchy consolidation, interstitial infiltrations, diffuse pulmonary oedema etc. Diffuse reticular opacity suspected of interstitial lung disease is considered as consolidation (not fibrosis).	Consolidation refers to an exudate or other product of disease that replaces alveolar air, rendering the lung solid	Ill-defined homogeneous opacity obscuring vessels, airway wall, probably with air bronchogram, without pull or push.
COVID-19	-	-	An algorithm which looks for signs of the novel Coronavirus (COVID-19) disease	*Suspected COVID-19*<br>No definition provided
Fibrosis	Scarring	Focal increased density in linear, curvilinear shape or branching pattern, suggestive of post-inflammatory scar lesion<br>It usually refers to post-inflammation scars. The most prevalent patterns are pleural thickening with fibrostreaky opacities shown on bilateral apex. Fibrosis is defined as post inflammatory fibrotic scar rather than fibrotic lung disease.	Any abnormal pulmonary fibrosis including interstitial fibrosis, fibrosis as part of fibro-cavitatory lesion or others (excluding pleural fibrosis).	Pulmonary scar<br>No definition provided
Hilar enlargement	Hilar abnormality (hilar tumour or lymph node enlargement)	-	An abnormality seen as hilar enlargement/ prominent hilum/ hilar lymphadenopathy	-
Mass (> 3 cm)	Mass > 3 cm	[Considered under nodule or mass]	-	Mass: over-3-cm
Mediastinal widening	Mediastinal widening above heart (mediastinal tumour or widening ≥8 cm at level of aortic knob)	Significantly enlarged mediastinal width at the aortic arch level or above<br>The intention of annotating this finding is to detect (1) aortic aneurysm/dissection; (2) significant paratracheal lymph node enlargement.	-	Widening mediastinum<br>Any signs of suspected widening of the mediastinum (e.g., aortic enlargement, widening of right paratracheal stripe, hilum overlay sign, cervicothoracic sign, enlarged pulmonary artery, mediastinal tumour, mediastinal lymph nodes, diaphragmatic hernia).

Medical device	-	-	-	Any medical instruments or devices used in patient support or treatment.
Nodule	Nodule (< 3 cm)	Nodule or mass<br>A round shaped, well or poorly defined pulmonary lesion. Here, nodule also includes mass (lesion size over 3 cm). Large mass that is highly suspected of lung cancer is also included.	A nodule is an abnormal rounded opacity seen in the lung parenchyma, well or poorly defined, measuring up to 3 cm in diameter	All ≤ 3-cm spots / nodular lesions on X-rays. Calcified nodules or nodules with a greater density than bone density: other findings
Oedema	-	-	-	Clear clinical cases, typical lesions on X-rays such as interstitial edema, bilateral perihilar edema, and batwing opacities.
Opacity	Opacity (consolidation, ground glass opacity, alveolar oedema)	-	Any abnormal focal or generalized opacity or opacities in lung fields (blanket tag including but not limited to consolidation, mass, infiltrate, nodule, calcification, interstitial thickening etc.)	This definition covers a wide variety of lesions which can be divided into the following types: air space opacification, linear opacification, and nodular opacification.<br>Typical cases of air space opacity, nodular pattern, reticular pattern, reticulonodular pattern, typical thickening of interstitial space à label lung opacity
Pleural effusion	Pleural effusion	An abnormal collection of fluid in the pleural space. In most cases, it will appear as costophrenic-angle blunting, but includes effusions in upper lung zones as well. In AP images, pleural effusion may appear as diffuse increased opacity in dependent portions.	Build-up of excess fluid around the lung in the pleural cavity	Uniform blurred cases, medium to high fluid volume, free-flowing pleural effusions with Damoiseau’s curve, typical subpulmonic effusions, typical encysted pleural effusions forming pseudo-tumor in patients with heart failure, etc.
Pleural other	-	-	-	Pleural thickening with divided edge and chest wall bone destruction at an unusual position of pleural effusion, clear calcification (cuttlefish form, along the pleura), also pleural tumours.
Pneumoperitoneum	Pneumoperitoneum	An abnormal collection of air in the abdominal space	Abnormal collection of gas or within the peritoneal cavity often due to a critical illness	-
Pneumothorax	Pneumothorax	An abnormal collection of air in the pleural space	Abnormal collection of air in the pleural space outside the lung	Most pneumothorax cases display such typical pictures as of avascular radiolucency, visible visceral pleura, deep sulcus sign, etc.

Rib fractures	-	-	Abnormal discontinuity seen with the rib borders most commonly as a sequelae of trauma.	All fractures visible on film (including old fractures), do not label bone destruction due to metastatic lesion, inflammation, etc.; do not label surgical-related bone loss. Clavicle, ribs, other fractures
Tuberculosis	-	Detects any suspicious regions of tuberculosis	An algorithm which looks for signs of pulmonary tuberculosis	Infiltration, nodular, cavernous, cavernous fibrosis, contractile in the upper half of the lung field, possibly on either or both sides. Chest X-ray images of people with HIV rarely show cavernous patterns; they often have interstitial tissue lesions, and probably in the lower areas of the lungs.

Other abnormalities considered by the expert radiologist: elevated hemidiaphragm, fibrosing interstitial lung disease, pulmonary hypertension, reticulonodular opacities, tracheal shift.

NOTE: Differences in output of CAD using Cardiomegaly as an example: qXR and DrAID rely on the cardio-thoracic ratio (CTR) of >0.5 for the diagnosis of cardiomegaly, while INSIGHT CXR defines this diagnosis as "significantly enlarged cardiac silhouette" without relying only on the CTR. In general CAD reports on abnormalities following a stepwise process by first localizing the region of interest (ROI), for cardiomegaly the hearts outline. Next, the severity of the ROI is assessed and depending on the CADs algorithm and definitions the cardiomegaly is classified e.g., by the overall silhouette, the CTR or the individual definitions of the developer. In addition to abnormality definition depending on the gold standard used during development including the population, setting and method. Therefore, having different outputs for the same abnormality in between different CAD tools.

Supplementary Table S3. Overview of diagnoses called as abnormal by the three CAD tools

Diagnosis	Number CXR included	INSIGHT CXR N (%) abnormal	qXR N (%) abnormal	DrAID N (%) abnormal
Atelectasis	30	30 (100)	30 (100)	28 (93)
Atypical pneumonia	30	29 (97)	30 (100)	29 (97)
Bronchiectasis	30	30 (100)	28 (93)	23 (77)
Cardiomegaly	30	27 (90)	27 (90)	24 (80)
COPD	30	29 (97)	29 (97)	25 (83)
COVID-19 pneumonia	16	16 (100)	16 (100)	16 (100)
Interstitial lung disease	30	30 (100)	30 (100)	30 (100)
Heart failure	30	30 (100)	29 (97)	30 (100)
Lung abscess	6	6 (100)	6 (100)	6 (100)
Lung cancer	30	29 (97)	29 (97)	27 (90)
Mediastinal lymphadenopathy	29	24 (83)	14 (48)	12 (41)
Metastases	29	28 (97)	29 (100)	27 (93)
Pleural effusion	30	30 (100)	30 (100)	30 (100)
Pleural thickening	8	8 (100)	8 (100)	5 (63)
Pneumocystis jirovecii pneumonia	30	30 (100)	29 (97)	29 (97)
Pneumonia	29	28 (97)	27 (93)	26 (90)
Pneumothorax	30	30 (100)	30 (100)	25 (83)
Pulmonary arterial hypertension	30	28 (93)	25 (83)	26 (87)
Sarcoidosis	30	29 (97)	28 (93)	24 (80)
Silicosis	10	10 (100)	10 (100)	10 (100)

COPD: chronic obstructive pulmonary disease; CXR: chest X-ray

Supplementary Table S4. Overview of detected abnormalities and scores for the three CAD

Abnormality detected	INSIGHT CXR 12 abnormalities		qXR 14 abnormalities		DrAID 19 abnormalities	
	Median score	N (%) positive	Median score	Proportion positive	Median score	Proportion positive
Abnormal	96.71	501 (97)	0.88	484 (94)	0.99	452 (87)
Atelectasis	4.06	142 (28)	-	-	0.07	139 (27)
Blunting (costophrenic angle)	-	-	0.47	95 (18)	-	-
Calcification	1.57	49 (10)	-	-	-	-
Cardiomegaly	1.54	145 (28)	0.32	118 (23)	0.06	129 (25)
Cavity	-	-	0.12	15 (3)	<0.01	32 (6)
Consolidation	77.95	370 (72)	0.47	220 (43)	0.09	154 (30)
COVID-19	-	-	-	-	0.67	281 (54)
Fibrosis	3.10	121 (23)	0.51	138 (27)	0.15	146 (28)
Hilar enlargement	-	-	0.64	16 (3)	-	-
Lung lesion	-	-	-	-	0.14	159 (31)
Mass	-	-	-	-	0.05	39 (8)
Mediastinal widening	0.76	42 (8)	-	-	0.05	76 (15)
Medical device	-	-	-	-	0.06	2 (<1)
Nodule	16.59	274 (53)	0.39	154 (30)	0.09	124 (24)
Oedema	-	-	-	-	<0.01	41 (8)
Opacity	-	-	0.92	428 (83)	0.99	353 (68)
Pleural effusion	2.70	151 (29)	0.31	97 (19)	0.07	116 (22)
Pleural, other	-	-	-	-	0.05	23 (4)
Pneumothorax	0.27	35 (7)	0.22	46 (9)	0.05	35 (7)
Pneumoperitoneum	0.24	4 (<1)	<0.01	1 (<1)	-	-
Rib fracture	-	-	0.03	5 (1)	0.05	27 (5)
Tuberculosis	5.36	126 (24)	0.29	150 (29)	0.07	12 (2)

"-" not called by the CAD tool

Supplementary Table S5. Tuberculosis called by CAD in CXRs from individuals with another clinical diagnosis

Diagnosis	Total X-rays	TB called by CAD, N (%)		
		INSIGHT CXR	qXR	DrAID
Atelectasis	30	3 (10)	6 (20)	0 (0)
Atypical pneumonia	30	9 (30)	15 (50)	1 (3)
Bronchiectasis	30	21 (70)	5 (17)	1 (3)
Cardiomegaly	30	0 (0)	0 (0)	0 (0)
COPD	30	8 (26)	6 (20)	0 (0)
COVID-19 pneumonia	16	6 (38)	10 (63)	0 (0)
Interstitial lung disease	30	5 (17)	10 (33)	2 (7)
Heart failure	30	1 (3)	6 (20)	0 (0)
Lung abscess	6	3 (50)	6 (100)	1 (17)
Lung cancer	30	13 (43)	14 (47)	0 (0)
Mediastinal lymphadenopathy	29	11 (38)	4 (14)	0 (0)
Metastases	29	4 (14)	9 (31)	0 (0)
Pleural effusion	30	4 (13)	8 (27)	0 (0)
Pleural thickening	8	1 (13)	2 (25)	0 (0)
Pneumocystis jirovecii pneumonia	30	7 (23)	13 (43)	0 (0)
Pneumonia	29	5 (17)	12 (41)	0 (0)
Pneumothorax	30	0 (0)	0 (0)	0 (0)
Pulmonary arterial hypertension	30	2 (7)	2 (7)	0 (0)
Sarcoidosis	30	17 (57)	13 (43)	3 (10)
Silicosis	10	6 (60)	9 (90)	4 (40)

Supplementary Figure S1. Example X-rays for different pathologies using the three CAD systems

**Diagnosis**	**INSIGHT CXR**	**qXR**	**DrAID**
Lung abscess	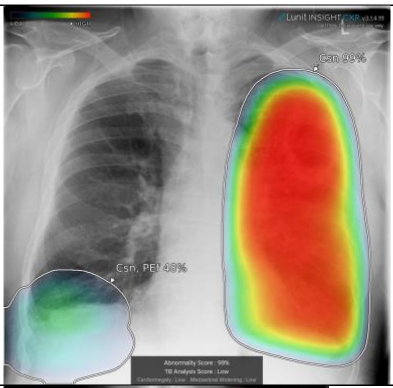	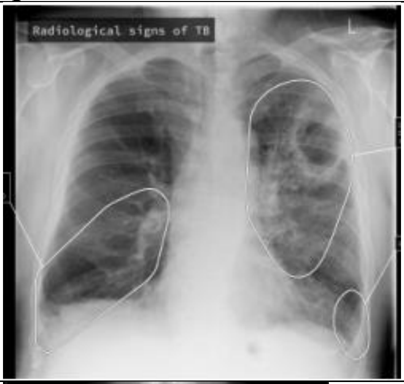	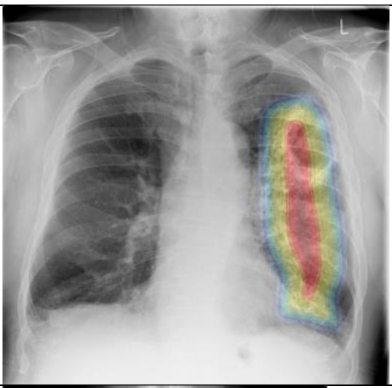
PCP	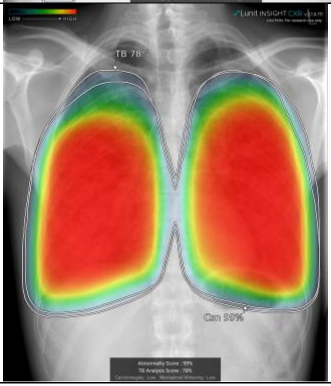	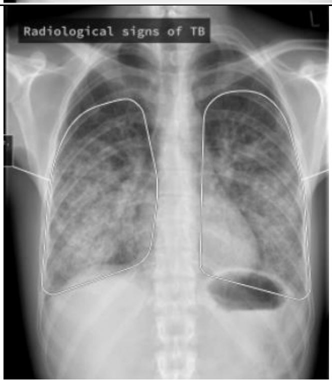	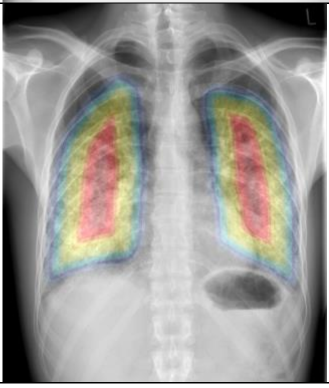

CXR: Chest X-ray; PCP: Pneumocystis jirovecii pneumonia

Supplementary Figure S2. Distribution of abnormality scores for INSIGHT CXR

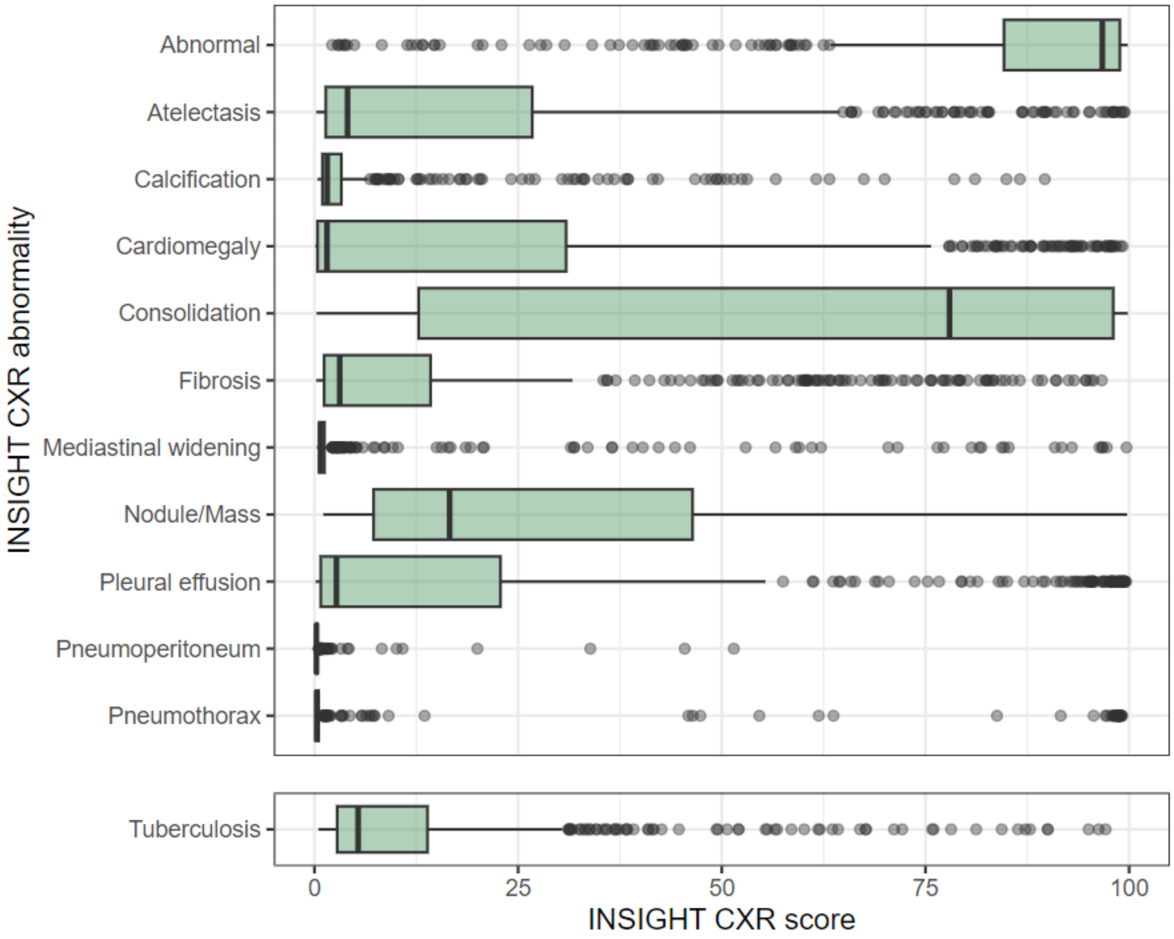

Supplementary Figure S3. Distribution of abnormality scores for qXR

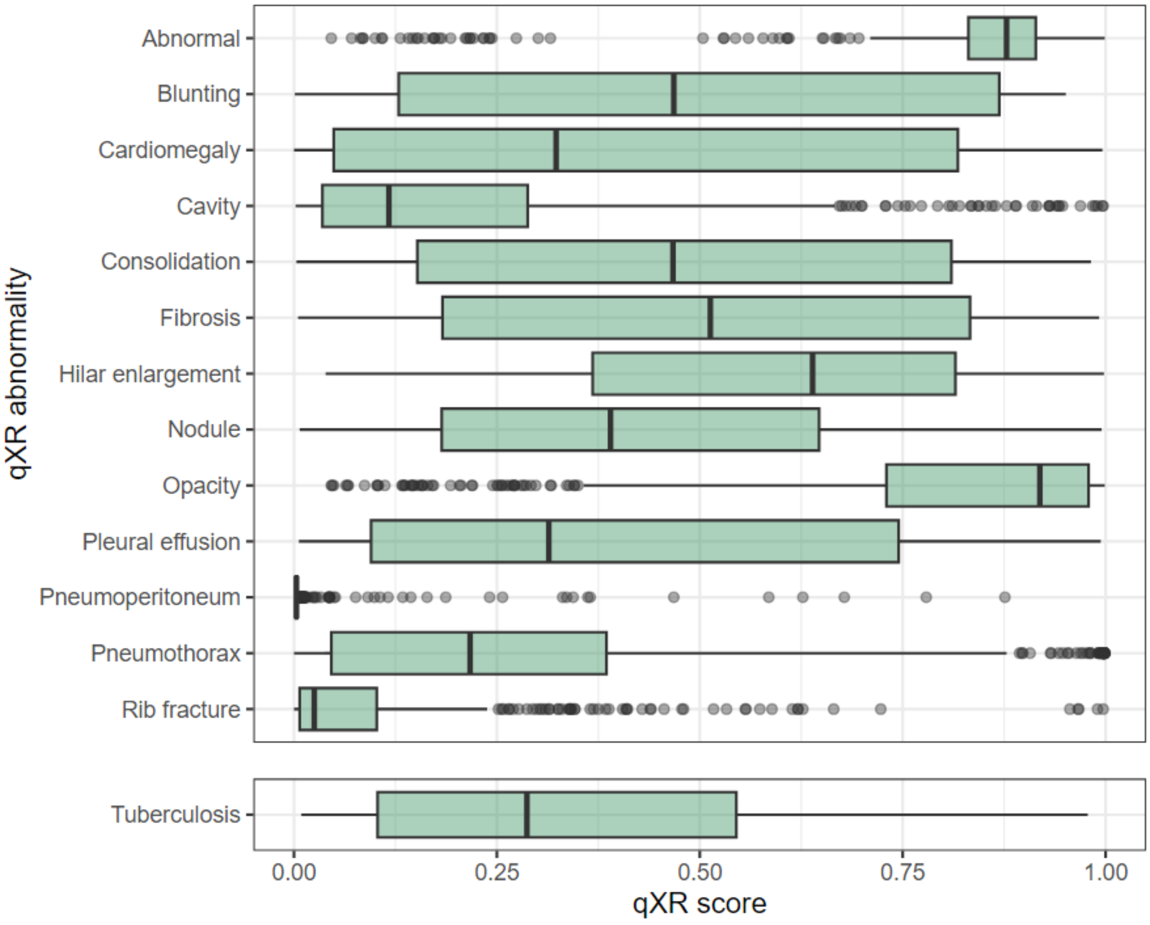

Supplementary Figure S4. Distribution of abnormality scores for DrAID

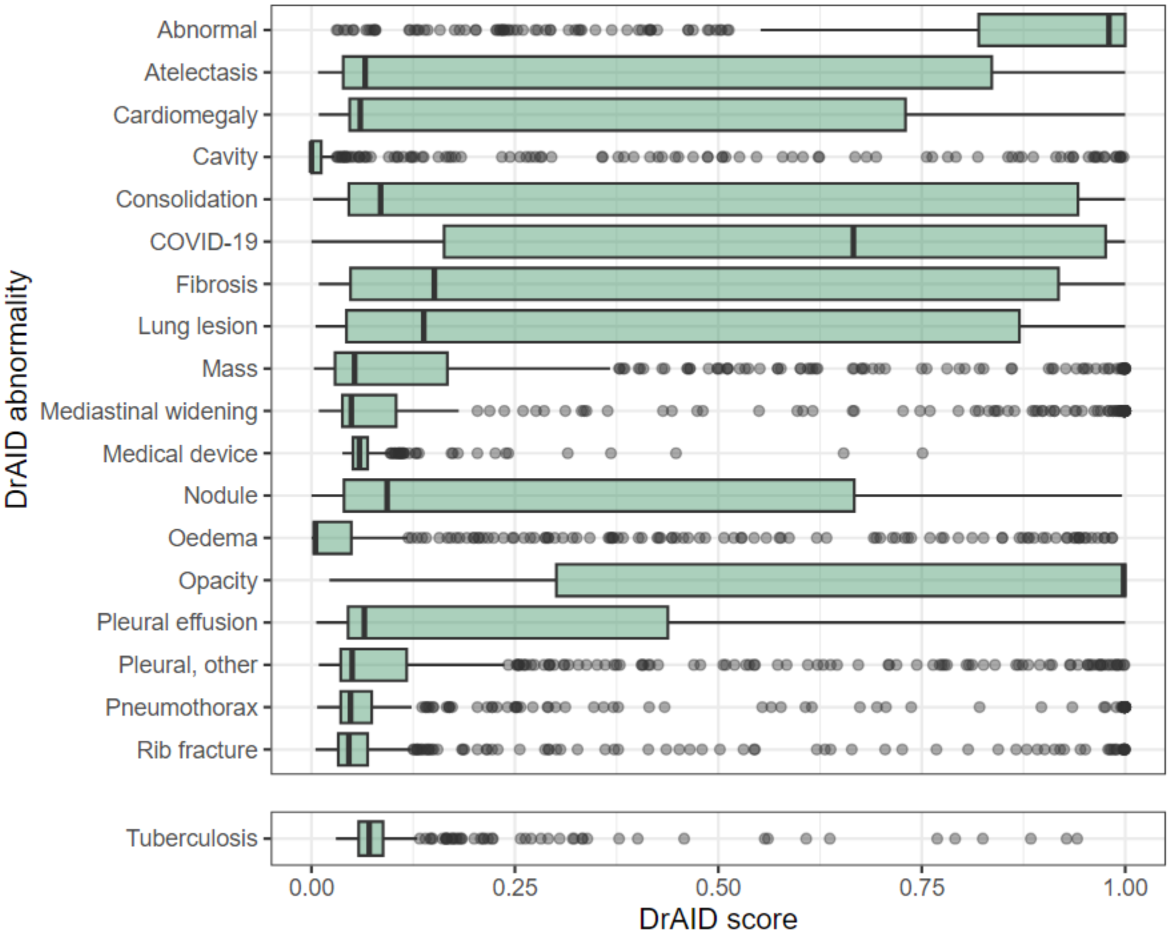

Supplementary Figure S5. Diagrams representing the overlap in reported findings for the three CAD technologies.

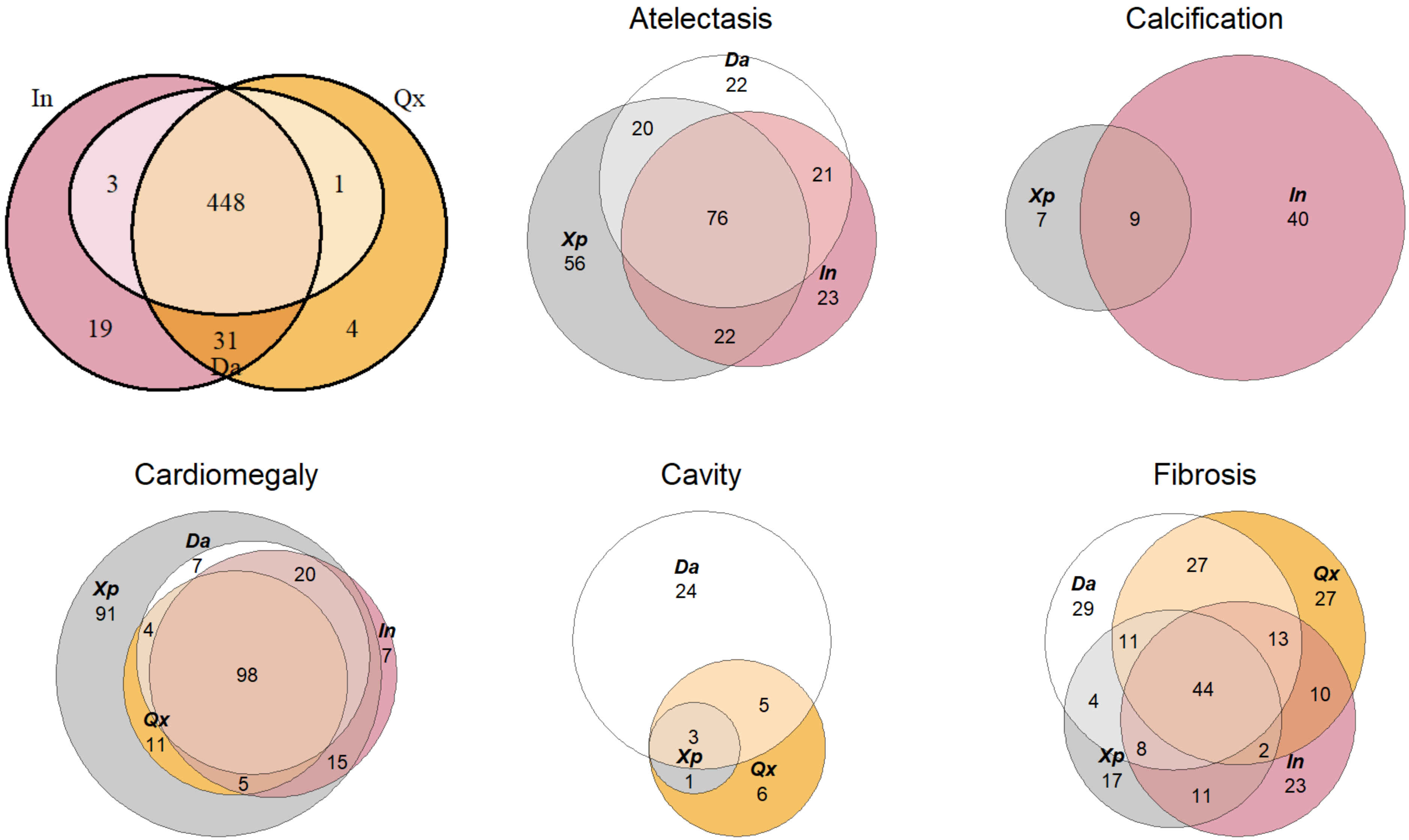

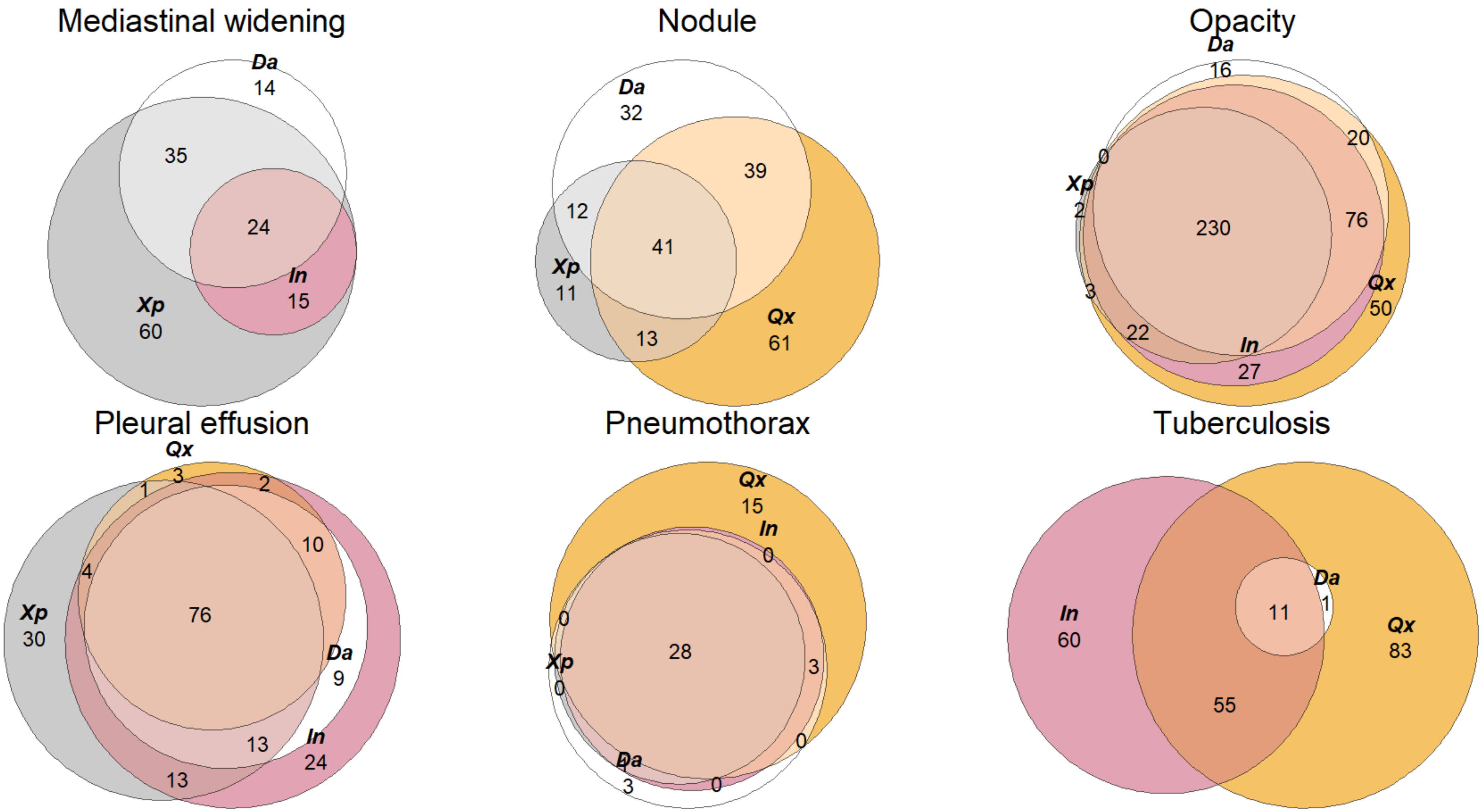

In: INSIGHT CXR, Qx:qXR, Da: DrAID, Xp: expert radiologist

Supplementary Figure S6. Receiver operator curves for the performance of the three CAD technologies in detecting different abnormalities

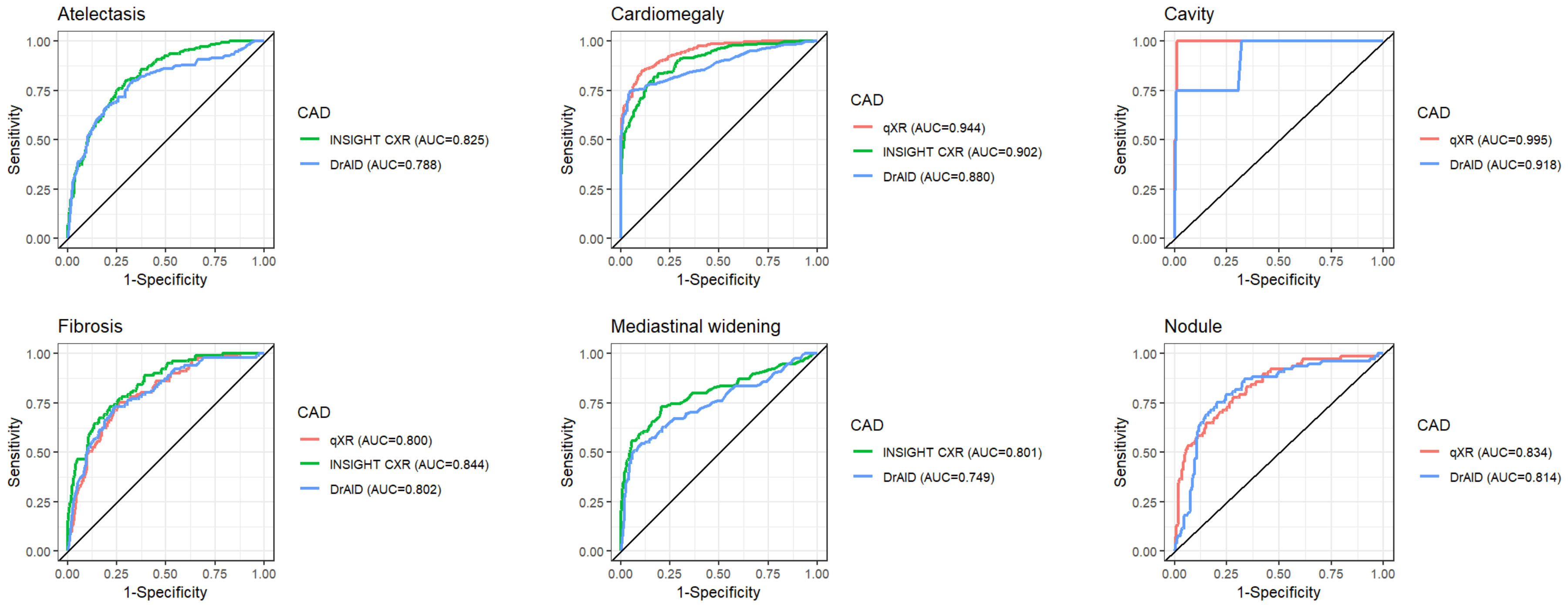

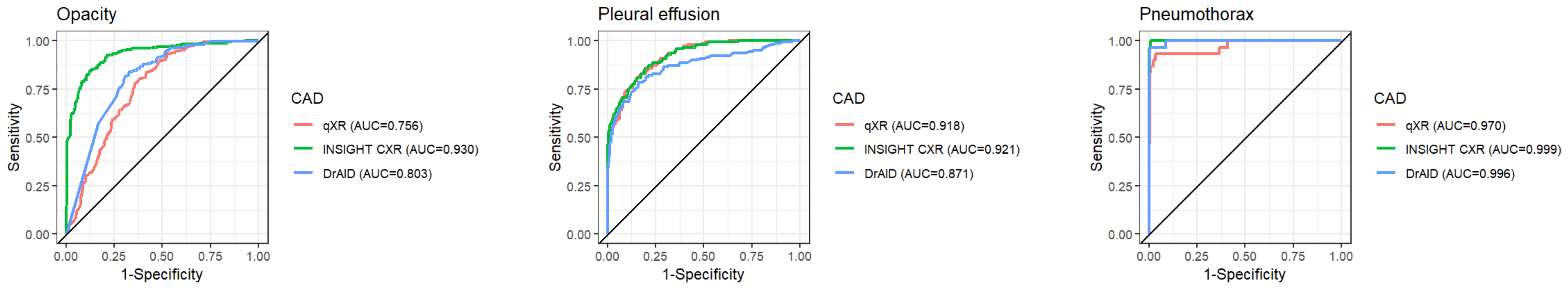
Opacity
Sensitivity
1-Specificity
0.00
0.25
0.50
0.75
1.00
CAD
qXR (AUC=0.756)
INSIGHT CXR (AUC=0.930)
DrAID (AUC=0.803)
Pleural effusion
qXR (AUC=0.918)
INSIGHT CXR (AUC=0.921)
DrAID (AUC=0.871)
Pneumothorax
qXR (AUC=0.970)
INSIGHT CXR (AUC=0.999)
DrAID (AUC=0.996)
